# Novel Hybrid Quadrupole-Multireflecting Time-of-Flight Mass Spectrometry System

**DOI:** 10.1021/jasms.2c00281

**Published:** 2023-01-05

**Authors:** Dale A. Cooper-Shepherd, Jason Wildgoose, Boris Kozlov, William J. Johnson, Richard Tyldesley-Worster, Martin E. Palmer, John B. Hoyes, Michael McCullagh, Emrys Jones, Robert Tonge, Emma Marsden-Edwards, Peter Nixon, Anatoly Verenchikov, James I. Langridge

**Affiliations:** † Waters Corporation, Stamford Avenue, Altrincham Road, Wilmslow, Cheshire, U.K.SK9 4AX; ‡ MSC-CG Ltd, Novi Bulevar A5, Bar85000, Montenegro

## Abstract

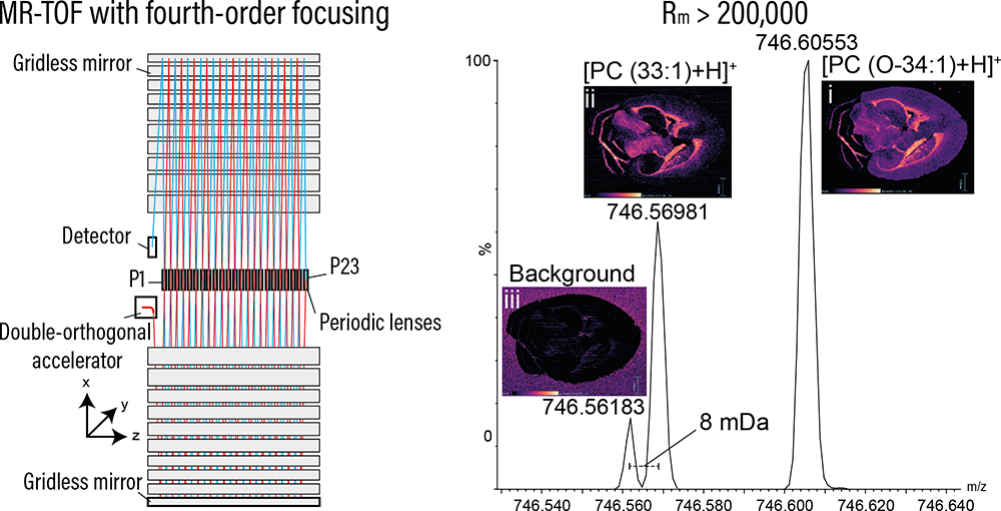

A novel mass spectrometry system is described here comprising a quadrupole-multireflecting time-of-flight design. The new multireflecting time-of-flight analyzer has an effective path length of 48 m and employs planar, gridless ion mirrors providing fourth-order energy focusing resulting in resolving power over 200 000 fwhm and sub-ppm mass accuracy. We show how these attributes are maintained with relatively fast acquisition speeds, setting the system apart from other high resolution mass spectrometers. We have integrated this new system into both liquid chromatography-mass spectrometry and mass spectrometry imaging workflows to demonstrate how the instrument characteristics are of benefit to these applications.

Time-of-flight mass spectrometry (TOF-MS) is a powerful technology in widespread use in the life, chemical, and physical sciences.^1,2^ In addition to its high sensitivity, high resolution, and mass accuracy, the approach is inherently fast with full-range mass spectra (say up to 1000 *m*/*z*) produced in tens to hundreds of microseconds from a single transient. TOF mass spectra are produced by summation over many such transients yielding acquisition speeds up to several hundreds of spectra per second.^3,4^ This speed is a key advantage over other high resolution MS approaches and has enabled TOF-MS to profile fast separation techniques such as rapid liquid chromatography (LC),^5^ gas chromatography (GC and GC × GC),^6^ capillary electrophoresis (CE), and ion mobility spectrometry^7,8^ (IMS) without compromising the fidelity of either separation. Furthermore, the speed of TOF-MS can ultimately enable a significant reduction in the overall duration of particular experiment types, for example, mass spectrometry imaging, where the time taken to generate an image is a function of the acquisition speed and spatial resolution.

With the range of benefits of TOF-MS, there are ever more efforts to further increase instrument performance, particularly resolving power and mass accuracy. The observed mass resolving power, *R*_*m*_, can be described in a simple equation:
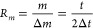
Where *m* is mass, *Δm* is the peak width in mass, *t* is the time-of-flight of an ion, and *Δt* its time width by virtue of spatial and velocity spread.

There are many strategies to increase resolving power in TOF-MS. One is to reduce *Δt* by minimizing the phase volume of the group of ions by collision cooling in a gas cell prior to the TOF.^9^ Another is to introduce energy-focusing ion mirrors in what are commonly known as “reflectron” TOF analyzers.^10^ A third strategy, often combined with ion mirrors, is to increase the accelerating voltage. This acts to reduce *Δt* by decreasing the ion packet turnaround time^1^ but has currently reached practical limits.

Reflectron TOFs are in widespread use and typically contain ion mirrors employing grids to homogenize the electric fields and provide energy focusing of the second order. In addition to energy focusing, reflectron TOFs have the added benefit of increasing the flight path length, increasing flight time, *t*, (and therefore *R*_*m*_) in a similar footprint to a linear TOF. The practicality of purely increasing flight length to improve *R*_*m*_ is met with considerations of analyzer size and instrument footprint and is often aided by the introduction of more passes of the ion packets through ion mirrors, introducing further “folds” in the flight trajectory (Figure 1).

**Figure 1:**
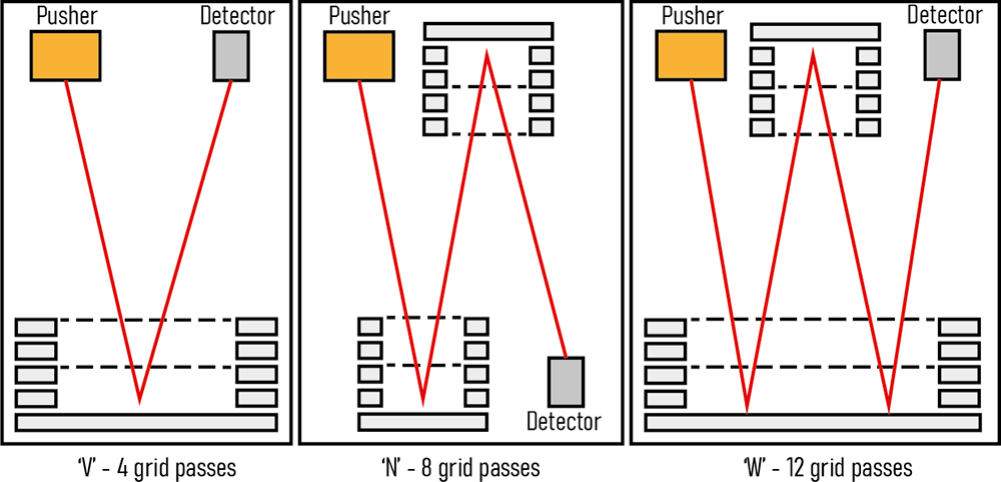
Three common TOF geometries. The “V” ion trajectory consists of four passes through mirror grids yielding optimal sensitivity-resolving power performance. In the “N” and “W” ion trajectories, resolving power is improved, but the number of grid passes (8 and 12, respectively) can affect overall sensitivity.

The grids in traditional reflectron analyzers have a transparency in the region of 90% due to geometrical ion losses and scattering. This means there is a significant cost in sensitivity as the number of passes through grids is increased. For this reason, the most prominent of high resolution TOF-MS analyzers comprise a “V” geometry with ions undergoing a single reflection. There are also commercial gridded analyzers with folded geometries in widespread use with so-called “N” or “W” flight paths,^11^ with the latter capable of resolving powers in excess of 100 000 fwhm.^12^ However, any further increase in the number of reflections requires further passes through mirror grids, affecting both sensitivity and duty cycle (Figure 1).

Many strategies for increasing flight length have been proposed including by the use of electrostatic sectors,^13−15^ segmented quadrologarithmic traps,^16,17^ and gridless mirrors.^18,19^ Gridless mirrors are used in so-called multireflecting (MR) TOF analyzers and come in two main families, closed-loop and open-loop. Closed-loop MR-TOF systems act as electrostatic traps and reflect ions back and forth over the same path. As a result, ion arrival times from ions having undergone different numbers of reflections can be similar, therefore restricting the mass range. These analyzers do, however, have widespread use in the analysis of small elemental species, in particular short-lived nuclides.^20^ In contrast, open-loop MR-TOF analyzers can capture the full mass range and have shown considerable promise in obtaining very high resolving powers without ion losses due to grid scatter. In particular, an MR-TOF with planar, gridless mirrors has shown significant utility in GC-MS applications.^4^ In analyzers of this type, periodic lenses are employed to mitigate ion beam divergence and control overall flight length, which has been extended up to 100 m and *R*_*m*_ up to 500 000 fwhm.^21^

Most commercial TOF analyzers operate with a “pulse and wait” scheme, where the time between pulses is set to match the time-of-flight of the greatest *m*/*z* in the range of interest. When analyzing a continuous ion beam, the pulse samples a small proportion of the incoming ions; until the next pulse, the continuous stream of incoming ions is allowed to continue straight through the acceleration region, remaining unsampled during the “wait”. With longer flight paths, the consequential increase in flight time necessitates waiting longer between pulses, leaving more ions unsampled. This describes a reduction in TOF duty cycle, which manifests as decreased sensitivity. In the limit of an extremely long flight length, the analyzer would spend so much time between pulses that its practical applications would be limited.

This reduction in duty cycle can be mitigated by multiplexing, that is, to remove the restriction of coinciding the pulse frequency with the highest *m*/*z* arrival time. This inescapably creates a situation where at any point in time the analyzer contains ions from multiple pulses producing an output signal that cannot be trivially calibrated. It has been shown that by encoding a sequence of time offsets into the pulse frequency, thereby operating with a pattern of pulses, rather than a fixed pulse period, the output signal can be successfully and reliably decoded back to one that accurately represents that of the individual pulses.^21^ This approach has been implemented commercially as Encoded Frequent Pushing^4^ in a 20 m Folded Flight Path GC-MR-TOF employing planar gridless ion mirrors with third-order energy focusing and capability of full spectrum resolving powers up to 25 000 (fwhm) in a compact geometry.^4^

TOF mass spectrometers have good mass precision even at moderate *R*_*m*_ as a result of their resolving power and high statistical profiling of ion signals resulting from sensitivity. By substantially increasing flight length, MR-TOF analyzers enforce a very high ratio between *t* and *Δt*, resulting in contributions to variation of *Δt* being negligible and leading to extraordinary mass accuracies in the sub-ppm range.^22^

Many workflows employing MS require high acquisition speeds, most commonly to adequately profile hyphenated chromatographic or electrophoretic separations. Fourier transform (FT)-based analyzers require long transient times (≥500 ms) to achieve high resolving powers, meaning for these systems, speed comes at the expense of MS resolution. There is, therefore, a need for systems capable of both high acquisition speeds and high resolving powers to accelerate these hyphenated approaches.

Mass spectrometry imaging (MSI) is a spatially resolved MS experiment that is increasing in popularity with the technique being applied to a wide variety of sample types.^23^ Determining the spatial localization of species in biological tissues remains the most impactful application of MSI, where the detection of changes to the distribution of biomolecules, drugs, and metabolites is central to understanding biological activity, disease, and pharmaceutical modes of action. There are several ionization sources used in MSI studies, the most common being matrix-assisted laser desorption ionization (MALDI)^24^ and desorption electrospray ionization (DESI).^25^ However, major challenges still exist in the confident identification of mass spectral signals and increasing the throughput of the analysis, the latter being an important consideration in medium to large cohort studies. Regarding mass measurement, as the nominal mass of a molecule increases, the number of chemical formulas potentially describing that mass also increases.^26^ This phenomenon makes mass accuracy the key metric in assignment confidence, which, in the absence of any upfront chromatographic or ion mobility separation, as in MSI, becomes all the more important, particularly for higher mass species.

In this Article, we describe a novel, high resolution, high mass accuracy quadrupole-multireflecting time-of-flight (Q-MRT) MS system. We outline the theoretical and technological bases of the new mass analyzer featuring an inclined double-orthogonal accelerator and planar gridless ion mirrors capable of fourth-order energy focusing. We demonstrate exceptional mass resolving power of greater than 200 000 (fwhm) and mass accuracy in the hundreds of ppb range all at acquisition speeds compatible with fast separations. The performance of the Q-MRT instrument will be discussed in the context of benefits to LC-MS and MS imaging applications.

## Instrument Design

### Overall Design

**Figure 2:**
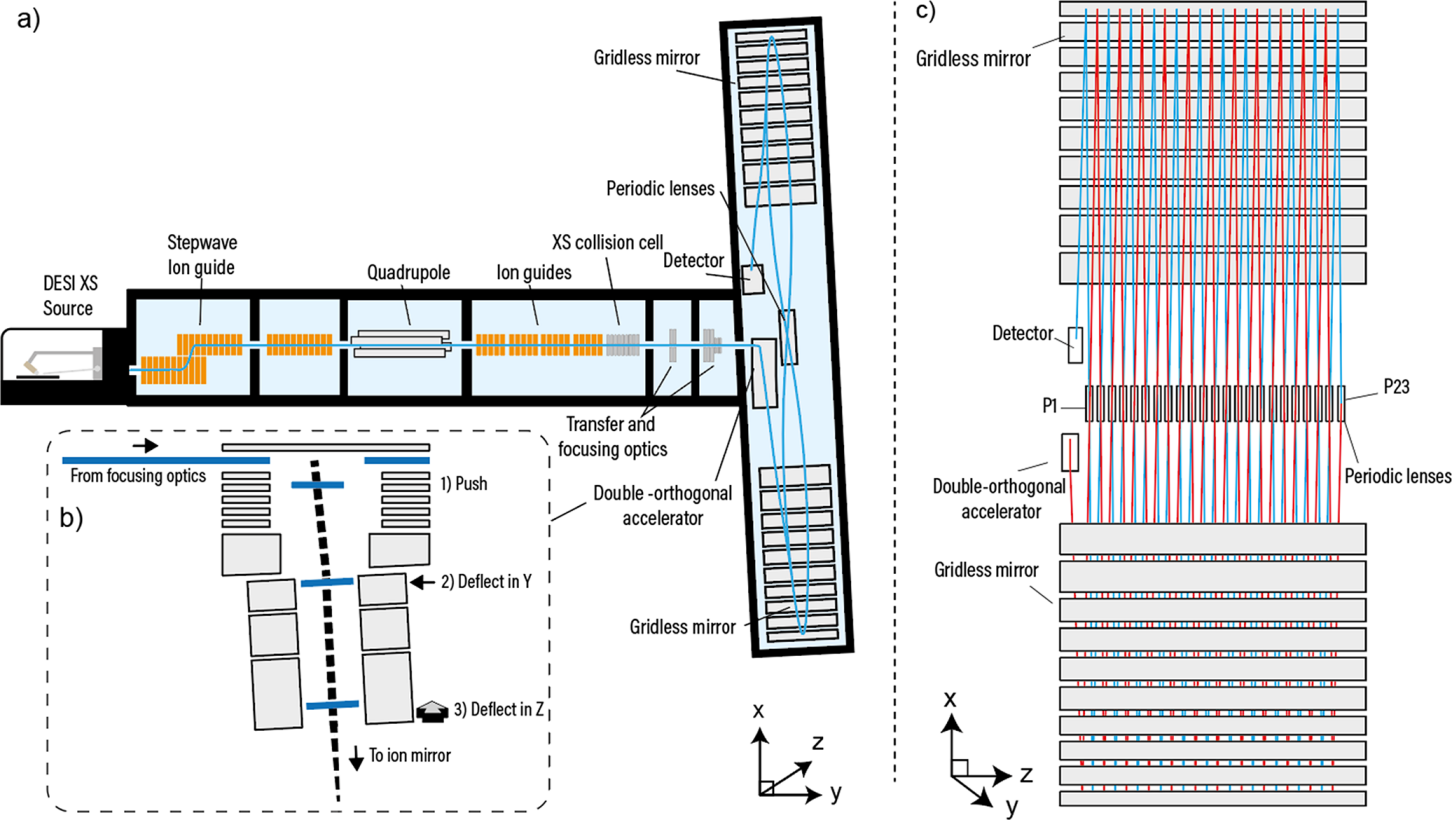
Schematic of the quadrupole-MRT instrument and the MRT analyzer. (a) The overall instrument design is “Q-TOF-like” with ions introduced in the source being focused onto the main axis via a StepWave ion guide and through a quadrupole followed by a segmented quadrupole collision cell. (b) Ions from the focusing optics are (1) pushed downward (−*X*) so that the combination of the push voltage and the ion’s +*Y* velocity results in a trajectory of 6° from vertical (*X* axis) . The mirrors are inclined in *X* at 3°, so the ion path is further rotated, at (2), with a retarding field in *Y*. This aligns the *X* path rotation with the center line of the mirrors. The ions are then deflected in *Z*, at (3), which provides the drift across the mirrors. After each reflection in *X*, the ions pass through a periodic lens, which compensates for beam expansion in *Z*. (c) The element P1 defines the ion beam angle into the mirrors, and P23 is arranged so that it reflects the ion beam back into the mirrors, which has the effect of doubling the flight length compared with positioning the detector at P23. In this arrangement, the effective flight length is ∼48 m. P1 can be operated independently to shorten the flight path (Figure S1).

The quadrupole-multireflecting time-of-flight system is represented schematically in Figure 2. Ions are sampled through a vacuum cone orifice into the first pumping stage of the instrument. Here the StepWave RF/DC ion guide captures ions and transfers them to a downstream quadrupole mass filter in the next vacuum stage, which enables selection based upon *m*/*z*. Positioned after the quadrupole is a transfer region made up of RF-confining DC stacked ring ion guides. Downstream of these guides is a gas-filled segmented quadrupole collision cell for collision-induced dissociation experiments and for conditioning the ion beam prior to reaching the transfer and focusing ion optics. After the focusing region is the multireflecting time-of-flight mass analyzer.

### MRT Analyzer

The employed MRT analyzer uses an open-loop approach and is distinguished from classical time-of-flight analyzers^27^ in three predominant ways; the double orthogonal acceleration and deflection of ion packets into the TOF, the gridless ion mirrors, and the periodic focusing of the ion packets during their time-of-flight separation. These attributes have been exploited previously on a GC-TOF-MS system from LECO Corporation^4^ with whom the authors have collaborated on the technology described herein. This GC-TOF system has a 20 m flight path made up of 32 reflections with ions of 1000 *m*/*z* having a flight time of approximately 600 μs. The mirrors provide third-order energy focusing, altogether affording a resolving power of 25 000 (fwhm) in a total analyzer size of 75 × 15 cm. In our implementation, the analyzer is larger (100 × 45 cm) with mirrors and lenses arranged to produce 46 reflections and a flight path of ∼48 m (and flight times of ∼1.3 ms for *m*/*z* 1000). In our case, the mirrors enable fourth-order energy focusing and an operating resolving power of >200 000 (fwhm). It should be noted that this is a flight length over an order of magnitude longer than most commercial TOF analyzers implemented in a similar overall instrument footprint. The underlying physics of the mirrors and an explanation of energy focusing in the context of TOF are described elsewhere;^28,29^ here we primarily describe our implementation and results.

### Orthogonal Acceleration and Deflection

A beam of ions exiting the collision cell is transferred to the orthogonal accelerator (OA) via a series of lenses. The application of voltage pulses accelerates packets of ions orthogonally, introducing an energy spread related to the size of the packets in the acceleration dimension (*X*) (Figure 2b). The initial velocity distribution and the acceleration field also define the turnaround time of the ion packets; both the turnaround time and energy spread are important parameters impacting *Δt* and hence TOF resolving power. Ions leaving the OA are deflected by parallel plates to compensate for ion velocity in the *Y* direction so that they can be directed into the mirrors. This *Y*-deflection introduces a 3° rotation of the ion packet in the *XY* plane matching its inclination to that of the mirrors and detector. After *Y*-deflection, the ions are then deflected in *Z* so that they traverse horizontally into the mirrors. This approach of double orthogonal acceleration produces ion packets that are relatively long (∼5 mm) in *Y*, increasing the sampling duty cycle, and relatively short (∼1 mm) in *Z* allowing multiple reflections to be packed in a short length as ions drift in the *Z*-dimension.

### Ion Mirrors

**Figure 3:**
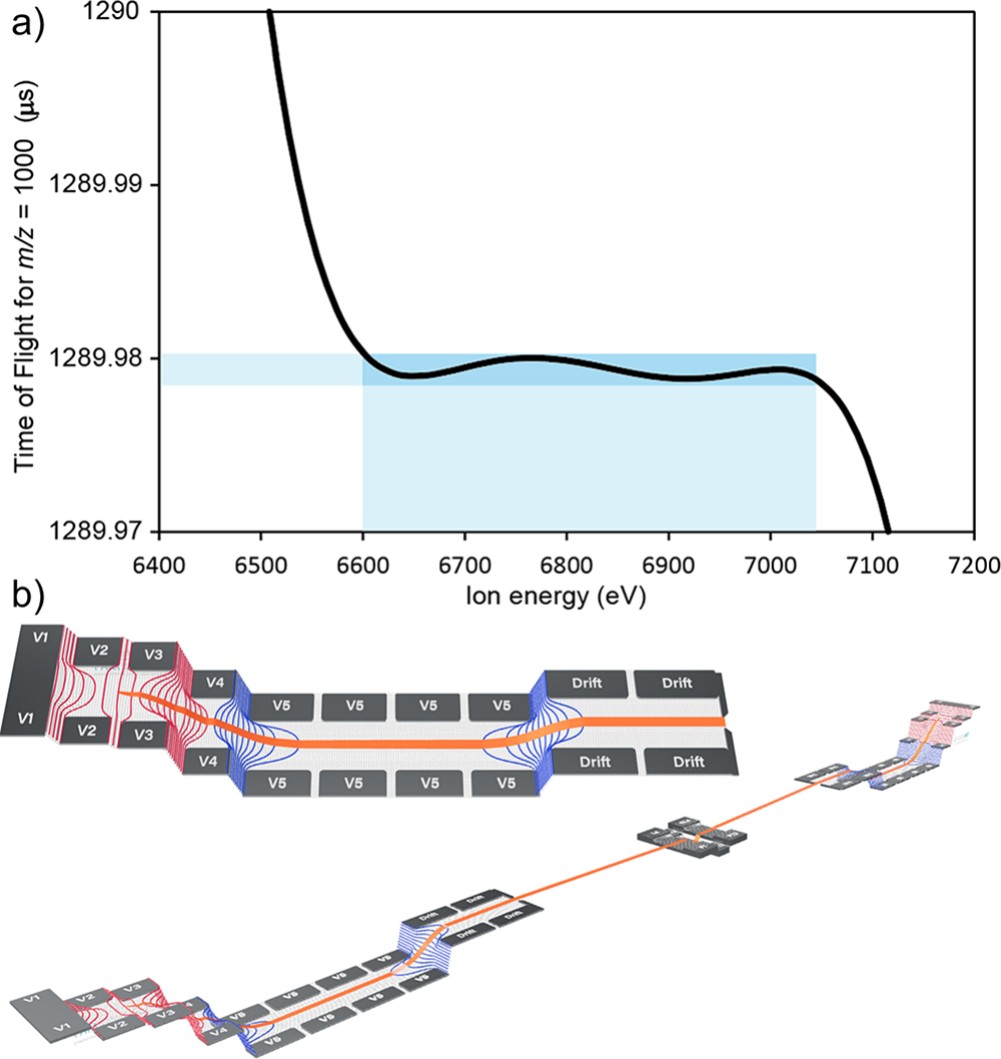
Energy focusing in the MRT analyzer. (a) Fourth-order energy focusing on the MRT enables a wide energy acceptance to facilitate high resolution separations. Ions with energies of 6600 to 7040 eV have flight times over a very narrow range of 1.5 ns (blue highlights). (b) Top: The MRT mirror potentials. From the drift potential, ions are accelerated into the mirror by potential V5. Potentials V4–V2 act to reflect the ion packets back through the mirror with V1 acting to better shape the reflecting potentials near the turning point. Bottom: Both mirror potential series can be observed in the context of the entire analyzer with the central periodic lenses visible between them.

After *Z*-deflection, the ions enter the first electrostatic ion mirror. The ion mirrors are gridless, which eliminates the ion losses associated with gridded mirrors. This is particularly important when considering analyzers with many reflections, as ion losses grow exponentially with the number of times the ion packets pass through the grids. The two ion mirror sets are extended in the drift direction (*Z*) and form a two-dimensional planar electrostatic field in *XY* (Figure 2c). The inhomogeneity in the electric fields of the OA and the ion mirrors, which is a consequence of the gridless approach, have been specifically arranged (through the design of the electrodes and the applied voltages) so that the aberrations are compensated at the end of the path, i.e., at the detector. This applies for both the temporal spread of the ion packet (*X*) and the spatial spread in *Y*. The electrostatic potential of the ion mirrors is shown in Figure 3b. To define these fields, five separate potentials (V1–V5) are applied to the 10 mirror electrode elements. Altogether, the energy focusing of the ion mirrors is of the fourth order^28^ (Figure 3a) where ions with energy from 6600 to 7040 eV have flight times almost independent of their energy. For a singly charged ion with mass 1000 Da, all flight times fit into a 1.5 ns variance with an average time of 1.28998 ms. This is sufficient to provide a theoretical mass resolving power of 500 000 (fwhm) in the absence of all other aberrations.

### Periodic Focusing

Ion packet divergence in the drift direction (*Z*) is controlled with an array of periodic focusing lenses located midway between the mirror sets (Figure 2a,c). The periodic lenses provide indefinite ion packet confinement along a zigzag ion trajectory allowing the flight path and flight times to be extended with minimal losses in sensitivity. These lenses introduce additional aberrations; however, these are moderate due to the small size of the ion packets in the *Z*-direction and the weak fields employed. The array consists of 23 lenses, with the distal lens (P23) being employed to reverse the ion packet drift motion in the *Z*-direction, extending the flight path by approximately a factor of 2. The first element (P1) is used to steer the flight path from the direction set by the exit lenses of the accelerator to the required path through the mirrors. It can also be set so that the flight path does not pass through the entire periodic lens array but makes a single reflection in each mirror before reaching the detector (Figure S1).

### Ion Detection

The extended flight times in the MRT analyzer enable the use of a detector with single ion pulse widths of ∼1 ns (fwhm) without degrading the effective resolving power, as the ion arrival spreads are still larger than this value. Likewise, the detector surface flatness and parallelism requirements are less severe than in short flight path analyzers. For these reasons, a 12 μm pore MCP Z-stack detector is used.

### Duty Cycle and Sensitivity

The ion packet dimension in *Y* is ∼5 mm. This is set by the operational characteristics of the OA and the practical mechanical dimensions of the mirrors. When utilizing an OA pulse cycle period of 2 ms, the sampling duty cycle of the analyzer is limited to ∼0.1–0.2% if we use the classical definition.^27^ To recover the sampling duty cycle, Encoded Frequent Pushing (EFP)^4,21,30^ is used with a 128 pulse pattern, significantly improving the sampling duty cycle to greater than 10%. This 128-fold increase in sensitivity improves ion statistics and extends the lower end of the dynamic range of the achievable sub-ppm mass accuracy on this analyzer.^4^ As well as improving the sampling duty cycle, the high frequency of pulsing utilized with EFP (which is 64 kHz, versus 8 kHz on a V-mode reflection TOF for a similar mass range) effectively spreads the incoming ion beam out in time, minimizing space charge effects within the TOF, which could affect mass accuracy and resolving power. Furthermore, the high pulse rate increases the time over which ions of the same *m*/*z* arrive at the detector reducing the likelihood of detector saturation issues and increasing the upper end of the dynamic range. Another benefit of the EFP method is the resulting file size. The EFP decoding algorithm recognizes the incoherent nature of chemical noise signals between transients and effectively removes it from the data. This noise reduction reduces file size significantly relative to standard TOF data. Some examples are given in the experimental.

## Experimental Section

### Reagents

Sulfadimethoxine was obtained as part of the Waters LCMS QC reference standard (part number 186006963). The TMT reagents were purchased from ThermoFisher Scientific and used to label the peptides present in the Waters MassPREP Digestion Standard Mix 1 (part number 186002865). l-Arginine (A5006) was purchased from Merck-Sigma.

### Separations and Direct Infusions

Sulfadimethoxine was separated from the components of the QC reference standard using an ACQUITY BEH 2.1 × 50 mm C_18_ column connected to an ACQUITY I-Class UPLC system. Mobile phases A and B were water and acetonitrile each with 0.1% (*v/v*) formic acid as additive. The percentage of B was increased from 2 to 90% over 2.2 min at 0.6 mL/min. The TMT128N/C reagents were used to tag MassPREP Digestion Standard Mix 1, and the resulting tagged digestion standards were diluted to approximately 1 μM in 1:1 acetonitrile:water with 0.1% (*v/v*) formic acid and mixed at a 1:1 ratio and introduced by direct infusion at 5.0 μL/min. Arginine was made up to 10 μM to effect cluster formation by electrospray and infused at 5 μL/min. An acquisition rate of 10 Hz was used for all of these compounds. The mass scale was calibrated for this application and the others presented using reference ions from sodium formate (0.5 mM) introduced by direct infusion in a solution of 90:10 2-propanol/water.

### Metabolite Identification Experiments

A sample of urine was obtained from a healthy volunteer 4 h post dose with carbamazepine (400 mg), acetaminophen (1000 mg), and naproxen (500 mg). The urine was diluted 10-fold with water prior to injection (10 μL) onto an ACQUITY HSS T3 C18 2.1 × 100 mm (40 °C, part number 186003539) column without any further cleanup. Chromatography was performed on an ACQUITY I-Class UPLC system. Mobile phases A and B were water and acetonitrile each with 0.1% (*v/v*) formic acid as additive. The percentage of B was increased from 1 to 15% over 3 min and then to 50% over 3 min at 0.5 mL/min. The column was washed and reconditioned over the remaining time giving a total run time of 12 min. Data were acquired in MS or MS^E^ mode employing leucine enkephalin at 556.276575 Da as lockmass. The file sizes for the 12 min runs were typically around 65 megabytes. All MS and LC-MS data were visualized and processed using Masslynx or UNIFI Software.

### DESI Profiling and Imaging

For DESI experiments, a DESI XS ion source was used. The spray solvent was 95:5 methanol/water with 100 pg/μL leucine enkephalin added to provide an internal lockmass. The spray flow rate was held at 2 μL/min. For the acquisition rate experiments, a 16 μm porcine liver section was prepared using a CM3050 CryoStat (Leica BioSystems). Acquisitions were performed over a 3 × 2 mm rectangular area with a 50 μm pixel size at 1, 2, 5, and 10 Hz lasting durations of 42, 22, 10, and 6 min, respectively, including row-to-row traverse times. Each resulting image contained 2400 pixels. The murine brain section was kindly donated by the Wolfson Molecular Imaging Centre, University of Manchester, UK. The DESI spatial resolution was set to 30 μm. The stage rate was 60 μm/s, and a modest acquisition rate of 2 Hz was employed yielding an image with 150 000 pixels in approximately 21 h. The filesize for this experiment was 3.9 gigabytes. Data were processed using Waters High Definition Imaging, HDI, software v1.6.

## Results

### Characterizing Resolving Power and Speed

**Figure 4:**
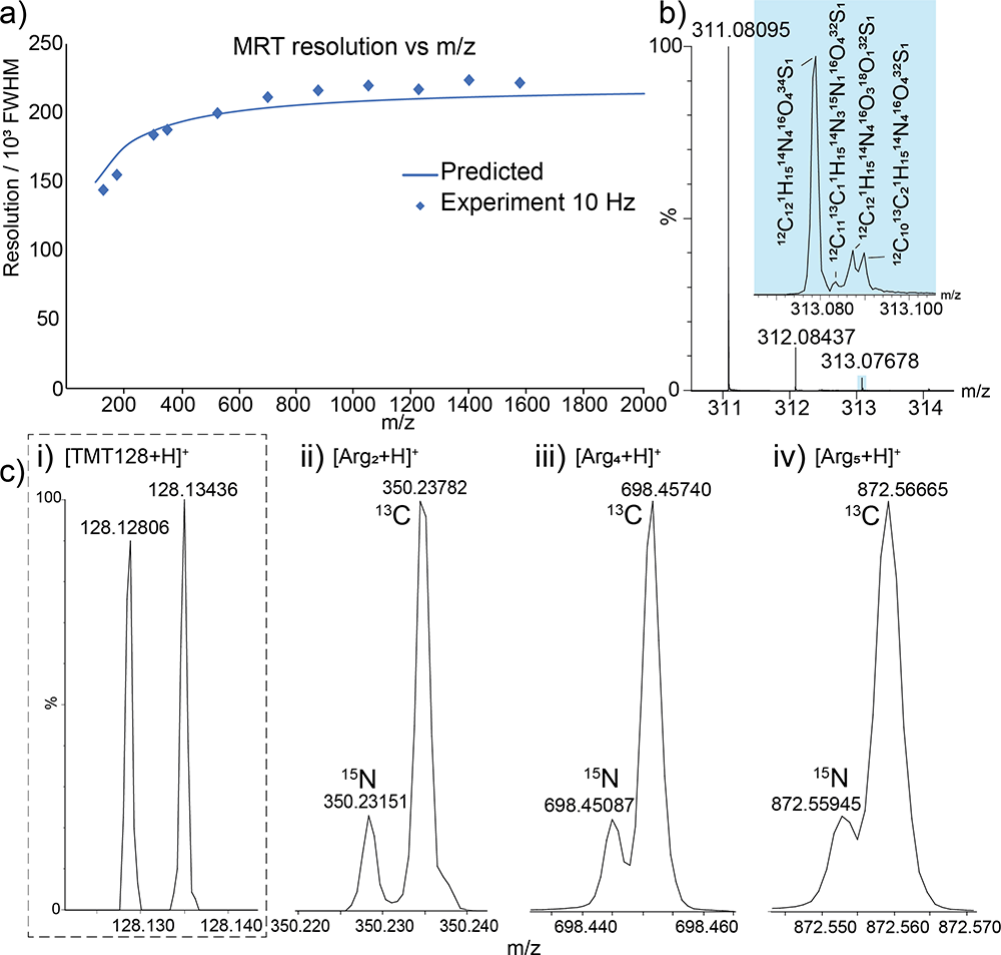
Characterizing resolving power. (a) The MRT analyzer exhibits resolving powers of greater than 200 000 (fwhm). (b) An LC-mass spectrum of sulfadimethoxine showing the fine isotope structure of the A+2 signal. (c) The MRT analyzer can distinguish the 6.3 mDa difference between the fine isotope signals of ^13^C and ^15^N up to approximately 900 *m*/*z* as shown here for clusters of arginine.

To explore the benefits of the new MRT analyzer, we plotted the achievable mass resolving power against *m*/*z* (Figure 4a). The predicted resolving power above 500 *m*/*z* is above 200 000 fwhm, dropping to 150 000 below 200 *m/z.* We acquired mass spectra for a number of compounds spanning the mass range 128 to 1600 *m*/*z*. The values of the resolving power are displayed on the plot showing the agreement between the predicted capability of the analyzer and that achieved experimentally. Another basis for evaluation of resolving power is the ability to distinguish particular fine isotope features (Figure 4b,c). Figure 4b shows a mass spectrum of sulfadimethoxine, a small molecule with rich isotope content. The signals exhibit a resolving power of above 180 000 (fwhm), which enables the distinction of ^13^C, ^15^N, ^18^O, and ^34^S signals in the A+2 isotope species. Furthermore, fine isotope structure is observed across a wide mass range; Figure 4c shows a collection of signals corresponding to separation of ^15^N from ^13^C, a difference of 6.3 mDa, for TMT128N and C (Figure 4c(i)) and a selection of clusters of the amino acid arginine (full mass spectrum in Figure S2). For the pair of TMT128 ions, the resolving power is above 140 000 (fwhm), which is sufficient to baseline separate and quantify these reporter ion species. The arginine cluster [Arg_2_+H]^+^ is measured with a resolving power of >190 000 (fwhm), whereas [Arg_4_+H]^+^ and [Arg_5_+H]^+^ both have resolving powers >200 000 (fwhm). The spectra show the A+1 signal from the isotope envelopes and demonstrate how the fine isotope structure is maintained up to approximately 900 *m*/*z*, which is unprecedented for a commercially available TOF system.

**Figure 5:**
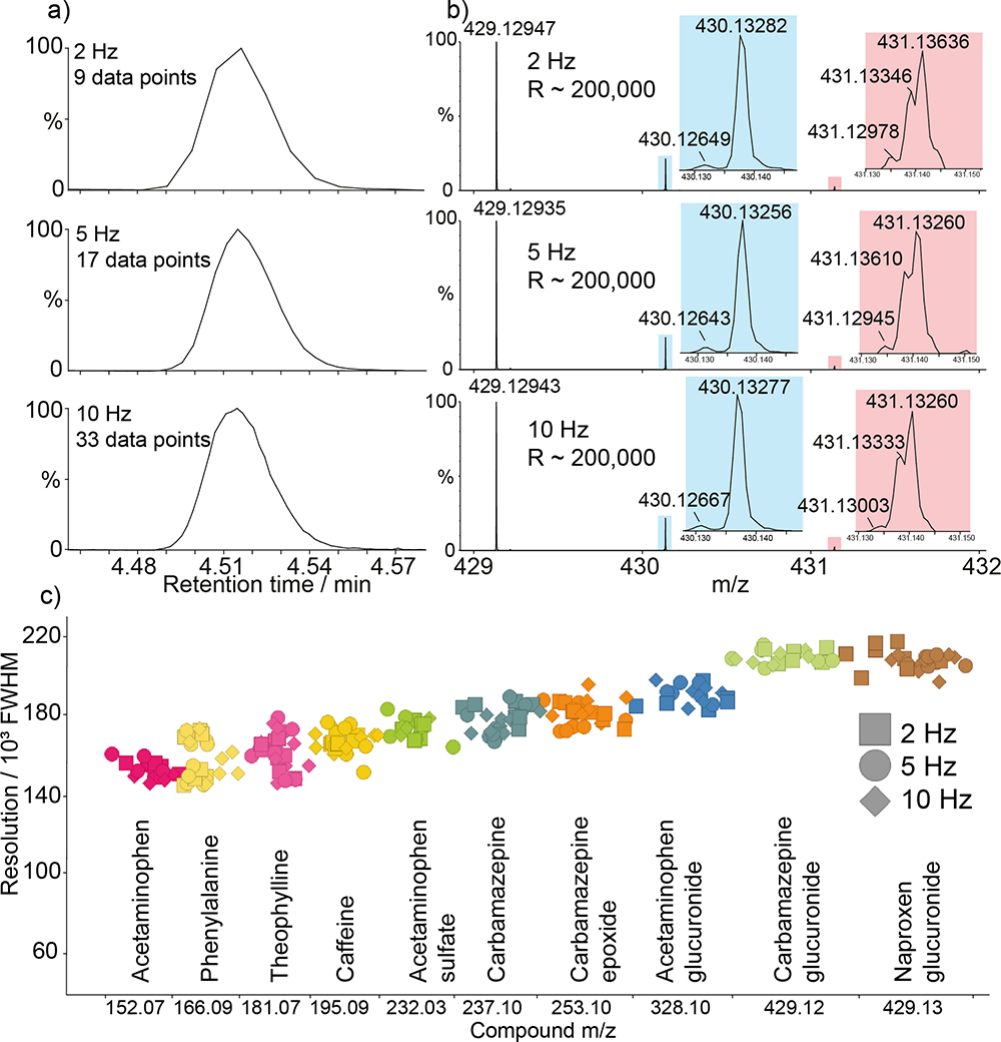
Resolving power at LC-MS speeds. (a) Extracted ion chromatograms for carbamazepine-*O*-glucuronide metabolite, illustrating the chromatographic integrity obtained at 2, 5, and 10 Hz. (b) Mass spectra of carbamazepine-O-glucuronide at 2, 5, and 10 Hz. Insets highlight the fine isotope structure of the A+1 (blue) signal with resolved ^12^C_21_^1^H_21_^14^N_1_^15^N_1_^16^O_8_ and ^12^C_20_^13^C_1_^1^H_21_^14^N_2_^16^O_8_ and A+2 (red) signals showing partially resolved ^12^C_20_^13^C_1_^1^H_21_^14^N_1_^15^N_1_^16^O_8_, ^12^C_21_^1^H_21_^14^N_2_^16^O_7_^18^O_1_, ^12^C_19_^13^C_2_^1^H_21_^14^N_2_^16^O_8_, and ^12^C_20_^13^C_1_^1^H_20_^2^H_1_^14^N_2_^16^O_8_ (all in increasing mass order) maintained at all speeds. (c) Plot of resolving power for urinary endogenous phenylalanine and xenobiotic compounds with detected metabolites acquired at 2 Hz (squares), 5 Hz (circles), and 10 Hz (diamonds).

To demonstrate the speed of the MRT analyzer in LC-MS workflows, we performed metabolite identification experiments on a sample of urine from a healthy patient. The Q-MRT instrument was operated at 2, 5, and 10 Hz acquisition rate with all other conditions held constant. A number of endogenous and xenobiotic species were identified (Figure 5). We selected a metabolite, carbamazepine-*O*-glucuronide, and interrogated its chromatographic profile and MS signals at the different acquisition rates (Figure 5a,b). At 2 Hz, the number of points across the LC peak was 9, which is less than that desired for accurate and precise quantification (10–12 points).^31^ At 5 Hz, 17 data points were recorded across the peak putting this in the range for very accurate quantification and confident characterization of potential coelution. At 10 Hz, the analysis achieved 33 data points across the peak. This latter case likely represents an oversampling of the chromatogram but demonstrates the potential in the MRT analyzer to profile even faster LC separations. Most notable here is that the observed resolving power is measured at ∼200 000 fwhm for the *O*-glucuronide at all acquisition speeds, revealing fine isotope structure in the A+1 and A+2 signals (Figure 5b), demonstrating that the performance of the MRT analyzer does not deteriorate with increasing acquisition speed as with FT-based analyzers. This is also important when considering product ion spectra (Figure S3) as the high resolving power (and mass accuracy) is maintained on the fragment species. Figure 5c shows a plot of observed resolving power versus identified compound for a number of endogenous and xenobiotic species detected in the urine samples at the different acquisition rates. Again, this plot demonstrates that the resolving power is independent of acquisition rate and holds over the mass range and highlights the possibility of rapid high mass resolution experiments. Furthermore, the dynamic range achieved in these experiments is up to 4 orders per second when using the EFP multiplexing method, which is considerable for the resolving powers obtained (Figure S4).

The MRT analyzer was also characterized with respect to large molecule analyses. Such applications, for example protein intact mass LC-MS, also benefit from increased resolving power, yielding resolved isotope distributions. Protein analysis applications may also benefit from the shortened flight path mode of operation, which yields isotope distributions amenable to accurate average mass determination with resolving powers on the order of 10 000 fwhm (Figure S5).

### Applicability to MS Imaging

Unlike LC-MS where the chromatographic separation dictates the sampling frequency and length of an experiment, the duration of MSI data acquisition is determined by the number of pixels in an image, in turn determined by the sample area and the spatial resolution.
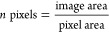


With one pixel being made up by a single mass spectrum, the faster the acquisition speed, the shorter the duration of the experiment. Speed in MSI workflows is of importance not just to increase sample throughput, but tissue section stability and MALDI-matrix volatility are also concerns. A recent study employing a 12 T FT-ICR mass spectrometer to localize oxaliplatin derivatives in human tissue demonstrated the utility of resolving powers of 200 000 (fwhm) at 457 *m*/*z* to confidently distinguish isobaric species.^32^ In this work, the FTMS system required an acquisition time of approximately 1 s. Also, a DESI-MSI study incorporating absorption mode processing (aFT) on a 7 T FT-ICR instrument also employed cycle times approaching 1 s for resolving powers approaching 160 000 (fwhm).^33^ Furthermore, a nano-DESI study utilizing a 21 T magnet with aFT processing required 768 ms transients to obtain resolving powers in the range of 300 000 (fwhm) for lipid species.^34^ Conversely, the MRT system, with comparable resolving power, has the potential to perform this type of analysis at much greater speed.

To investigate the experimental mass resolving power in the context of MSI, we performed DESI imaging experiments on small 3 × 2 mm portions of a 16 μm porcine liver section at acquisition speeds of 1, 2, 5, and 10 Hz. The mass spectra (Figure S6) show signals exhibiting the high resolving power of the MRT for a collection of lipids. In these spectra, we observe fine isotope structure at a nominal mass of 798 Da for all tested acquisition speeds, demonstrating how resolving power is maintained at all rates. We made four putative identifications based on the signals observed in the 40 mDa window as they were consistent with three different phosphatidylcholines PC (34:1) [M+^39^K]^+^; PC (34:2) [M+^13^C_2_+^39^K]^+^, (34:2) [M+^41^K]^+^; and a phosphoserine PS(O-36:1) [M+Na]^+^. The ion images for PC (34:1) [M+^39^K]^+^ are shown in Figure S6(b) and show a consistent image quality at all acquisition speeds.

**Figure 6:**
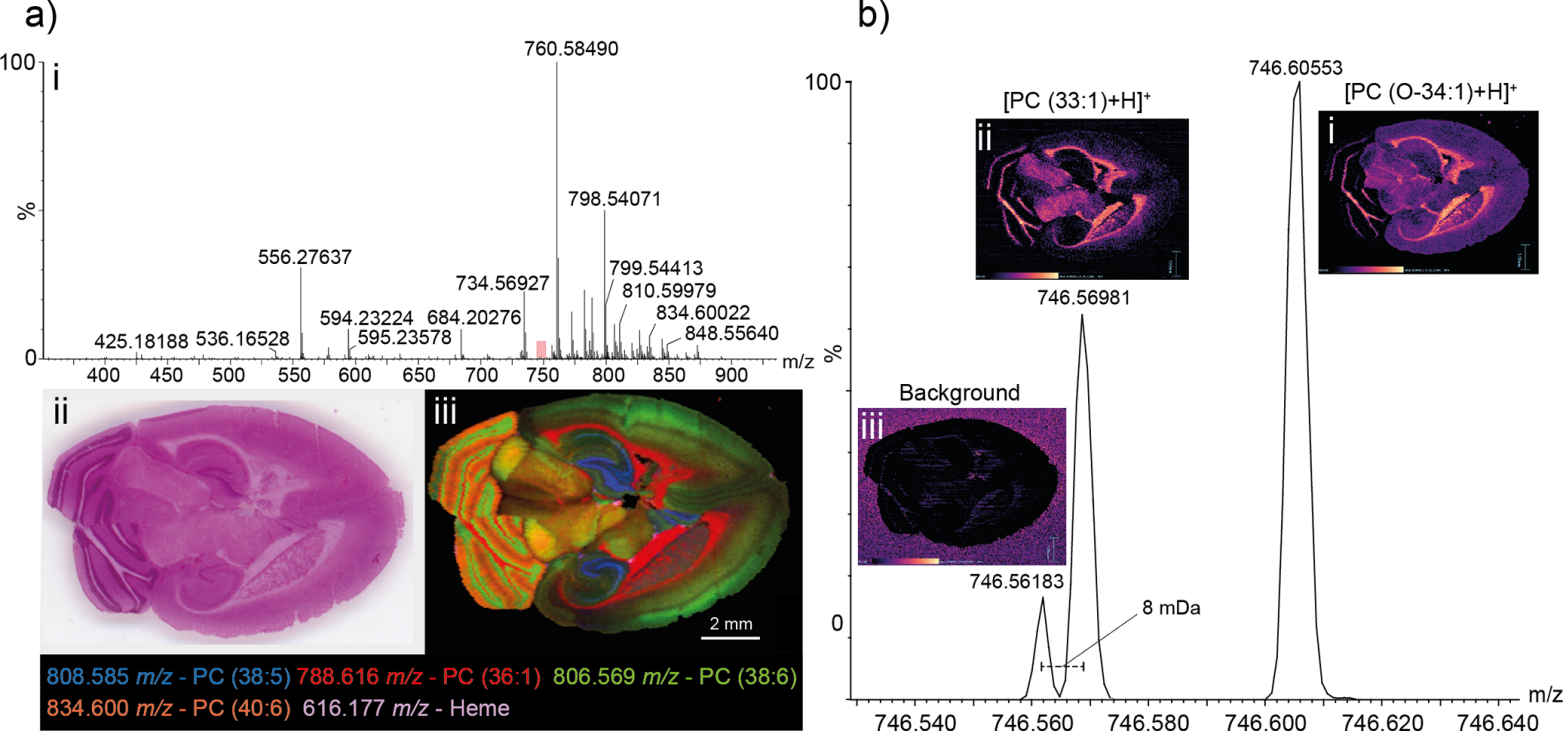
DESI-MSI analysis of murine brain. (a) A representative DESI mass spectrum (i) obtained as part of an MSI experiment on the murine brain. The lipid envelope is observed between 700 and 900 *m/z*. The murine brain section, shown as an H and E stained optical image (ii) was imaged in its entirety. A selection of identified species is shown in an overlaid molecular image (iii). Signals correspond to putative identifications of PC (38:5) (808.58542 *m/z*, blue, 420 ppb), PC (36:1) (788.61609 *m/z*, red, −370 ppb), PC (38:6) (806.56909 *m/z*, green, −423 ppb), PC (40:6) (834.60034 *m/z*, coral, 469 ppb), and Heme (616.17682 *m/z*, violet, 101 ppb). (b) Demonstration of the power of the high resolution MRT for imaging. A lipid signal corresponding to PC (33:1) is well-resolved from nearby lipid species but also from interfering background. On a lower resolution system, the background would interfere with the PC (33:1) signal yielding a composite image.

To further assess image quality for the Q-MRT instrument, we performed a DESI imaging experiment on a transverse murine brain section at 30 μm spatial resolution in positive ion mode (Figure 6). The DESI mass spectrum exhibited a range of signals with a predominant envelope between 700 and 900 *m*/*z* corresponding to lipid species desorbed from the tissue surface. A composite ion image of several detected species was constructed showing localizations to different brain tissues. The signal at 808.585 *m*/*z* is putatively identified as PC (38:5) with a mass error of 280 ppb and is localized to the hippocampal formation (blue). The signal at 788.616 *m*/*z* (red) locates primarily to the striatum and to the cerebellum; the signal at 806.569 *m*/*z* (green) locates to the cerebral cortex and cerebellum; and the signal at 834.600 *m*/*z* (coral) to the cerebellum. These signals are putatively identified as PC (36:1) (H^+^, 370 ppb), PC (36:4) (Na^+^, 423 ppb), and PC (40:6) (H^+^, 469 ppb), respectively. Signals for heme at 616.178 *m*/*z* (violet) are visible in small regions throughout the section thought to be blood vessels.

The resolving power afforded by the MRT analyzer allows the generation of molecular images for nominally isobaric species that are very close in mass. In Figure 6b, we present a mass spectrum over a narrow 100 mDa region. The major signal at 746.60553 *m*/*z* ([PC (O-34:1)+H]^+^, 384 ppb) is well resolved from the others in the spectrum and yields an image with localization to the cortex, midbrain, and fibrous regions of the striatum and cerebellum. A second lipid is observed at mass 746.56981 *m*/*z* ([PC (33:1+H]^+^, 500 ppb) and is baseline resolved from a signal a mere 8 mDa away in mass at 746.56183 *m/z,* which (due to its localization off-tissue) is thought to be nonendogenous background related species. Lower resolving power systems would produce composite images in this case, demonstrating the utility of the Q-MRT for imaging applications.

### Mass Accuracy

The extended flight path of the MRT analyzer imposes a high ratio between flight time, *t*, and time spread, *Δt.* As such, the analyzer is capable of very high mass accuracy in the hundreds of parts-per-billion range. To demonstrate this, we took a sample of ions from both positive and negative ionization modes from the DESI-MSI data of the murine brain and made identifications based on the observed *m*/*z* values (spectra shown in Figures S7 and S8). The list of identifications is shown in Tables S1 and S2. In positive ion mode, the identifications were mostly lipids in the range 369 to 878 *m*/*z* and yielded an RMS mass error of 386 ppb. As an example of the mass accuracy distribution across the imaging experiment we have plotted the observed mass error for four ubiquitous lipids in 50 spectra spaced throughout the positive mode run (Figure S9). The standard deviation for the mass error values was observed to be around 400 ppb for each reported lipid. In negative mode, the identifications spanned the range 115 to 885 *m*/*z* and included lipids, fatty acids, and small metabolic acids and yielded an RMS mass error of 357 ppb. These levels of mass accuracy are important in making confident identifications in particular for imaging applications where upfront chromatographic separations are not possible.

## Concluding Remarks

Here we have described the design and performance characteristics of a novel hybrid quadrupole-multireflecting time-of-flight mass spectrometer. The MRT analyzer enables fast acquisition of mass spectra with high resolving power >200 000 (fwhm) that is independent of acquisition speed and does not deteriorate with increasing mass-to-charge. These attributes set it apart from other high resolving power systems in particular those incorporating trapping, FT-based analyzers. The resolving power is afforded by an extended flight path TOF employing planar gridless ion mirrors with fourth-order energy focusing. We have demonstrated that the Q-MRT system is capable of distinguishing fine isotope detail of masses up to approximately 900 *m*/*z* and that the system shows significant utility in LC-MS and MSI applications, providing exceptional image clarity in the latter. Another key feature of the analyzer is the sub-ppm mass errors, which are typically associated with ultrahigh resolving power FT-ICR systems.

## Supporting Information Available

Schematic showing the shortened flight path mode of operation, supporting spectra, tables of identifications, and mass error versus time plots from the 21 h tissue imaging experiments (PDF)
