# Supplementary material for: Novel Hybrid Quadrupole-Multireflecting Time-of-Flight Mass Spectrometry System
Source: J Am Soc Mass Spectrom. 2023 Jan 5;34(2):264–72. doi: 10.1021/jasms.2c00281 (PMC9896552; doi:10.1021/jasms.2c00281)
Supplement: Supplementary file 1 — js2c00281_si_001.pdf [file js2c00281_si_001.pdf]

Supplementary Information

A novel quadrupole multireflecting time-of-flight mass spectrometry system

Dale A. Cooper-Shepherd[1], Jason Wildgoose[1], Boris Kozlov[1], William J. Johnson[1], Richard Tyldesley-Worster[1], Martin E. Palmer[1], John B. Hoyes[1†], Michael McCullagh[1], Emrys Jones[1], Robert Tonge[1], Emma Marsden-Edwards[1], Peter Nixon[1], Anatoly Verentchikov[2], James I. Langridge[1*]

1. Waters Corporation, Stamford Avenue, Altrincham Road, Wilmslow, Cheshire, UK, SK9 4AX

2. MSC-CG Ltd, Bar, Montenegro

† Present Address: TrueMass, Lower Harthill Barn, Goddard Lane, Rowarth, High Peak, Derbyshire, UK, SK22 1ED

Table of Contents

Figure S1. The MRT analyzer operated with a shortened flight path. S2

Figure S2. MRT mass spectrum of L-arginine clusters across the mass range 175- 1569 *m/z*. S3

Figure S3. Carbamazepine-o-glucuronide product ion spectrum. S3

Figure S4. Experimentally observed intra-spectrum dynamic range for the Q-MRT instrument. S4

Figure S5. A comparison of the ‘diamond’ and full flight path modes of operation. S5

Figure S6. The capabilities of the MRT analyzer for MSI. S6

Figure S7. Positive ion DESI mode spectrum from mouse brain tissue S7

Figure S8. Negative ion DESI mass spectrum from mouse brain tissue S9

Table S2. Mass accuracy in negative ion DESI mode S10

Figure S9. Mass accuracy for selected evenly-distributed lipids across a DESI-MSI experiment performed on murine brain. S12

Supplementary Methods S13

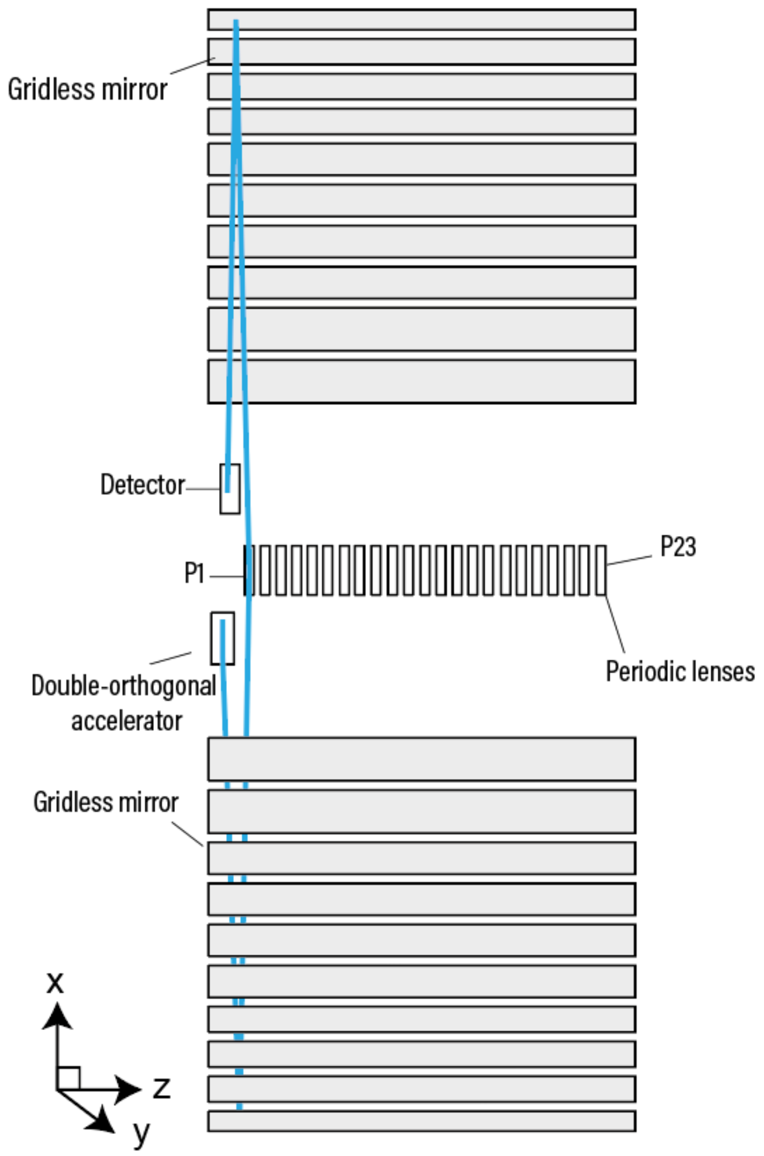

Figure S1. The MRT analyzer operated with a shortened flight path. The double orthogonal accelerator deflects ions into the first mirror and into lens P1. Unlike in full flight path mode P1 deflects the ion packet into the second mirror and to the detector. Altogether the flight path describes a ‘diamond’ shaped flight path. The operating resolving power of this mode is of the order of 10,000 FWHM.

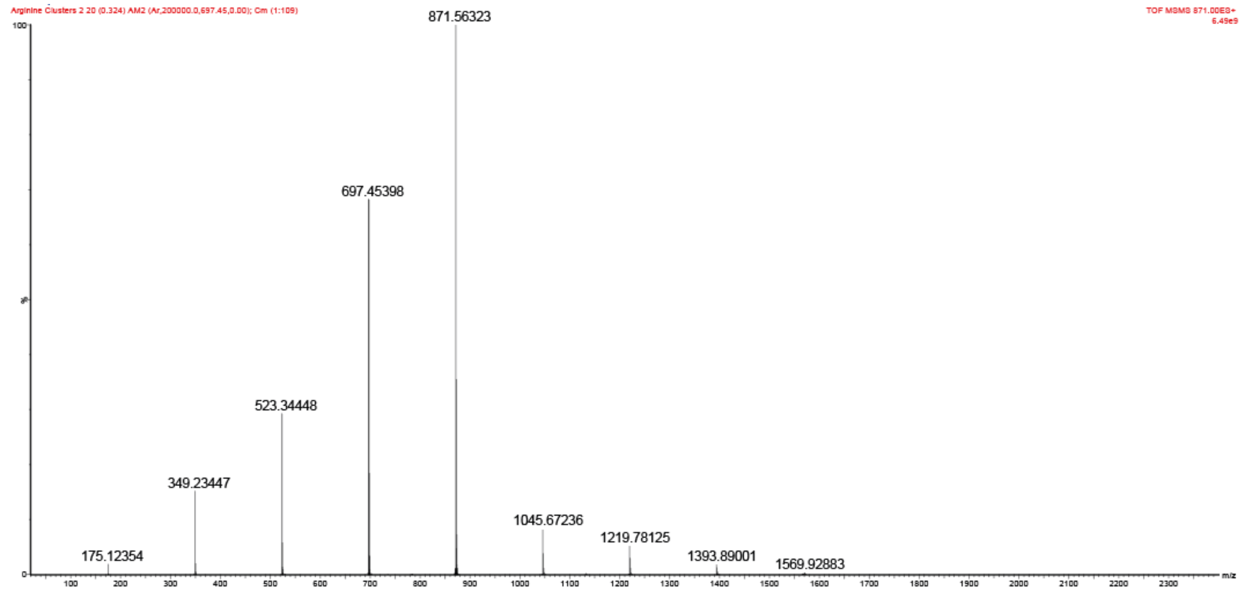

Figure S2. MRT mass spectrum of L-arginine clusters across the mass range 175- 1569 *m/z*.

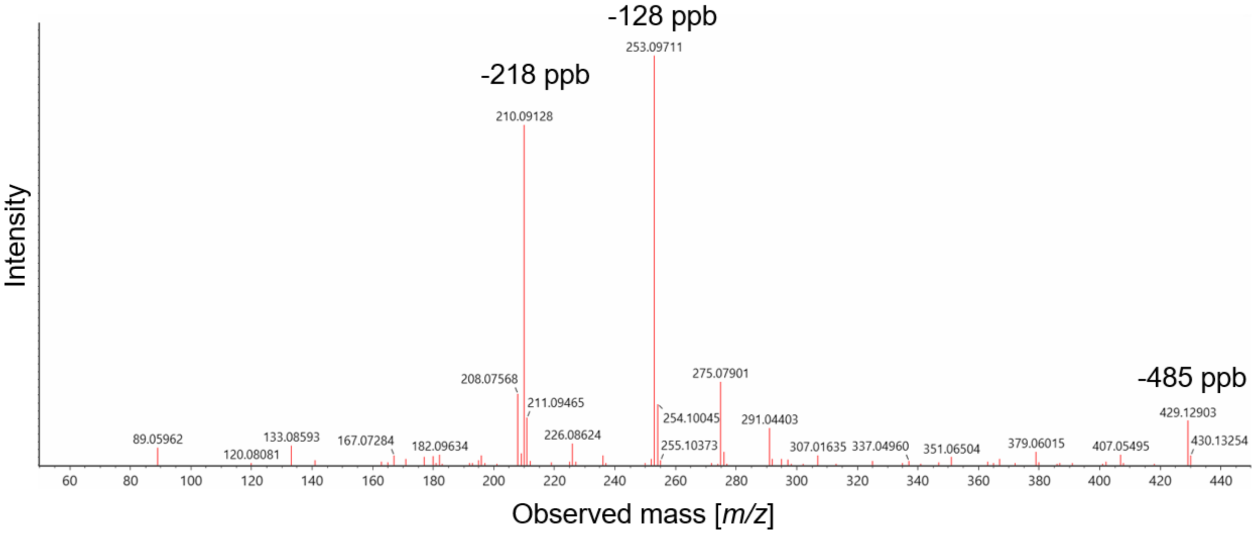

Figure S3. Carbamazepine-o-glucuronide product ion spectrum. The observed mass resolution on the product ions is the same as for precursors of the same mass. Observed mass errors are shown above the signals.

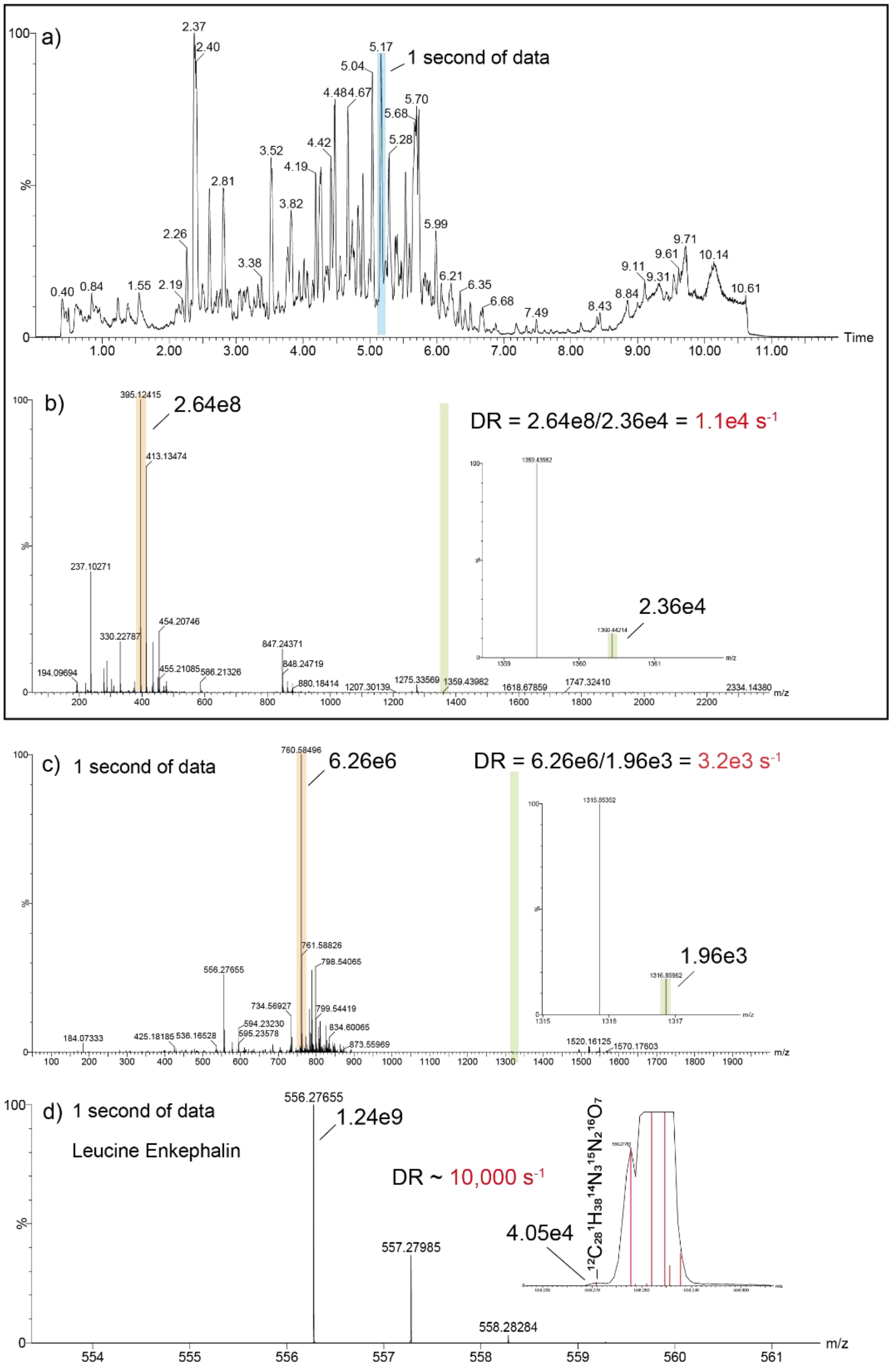

Figure S4. Experimentally observed intra-spectrum dynamic range (DR) for the Q-MRT instrument. a) total ion chromatogram for a single run from a metabolite identification study. A spectrum from one second of data was taken from one of the intense peaks in order to assess the dynamic range. b) By taking the observed intensity of the base peak and a low lying signal the dynamic range was calculated to be 1.1e4 s^{-1}. c) One second of data taken from a DESI imaging experiment. The dynamic range of this spectrum was measured to be 3.2e3 s^{-1}. d) A quantitative assessment of dynamic range using fine isotope signals from Leucine enkephalin. The signal from the fine isotope species $^{12}C_{28}{}^{1}H_{38}{}^{14}N_{3}{}^{15}N_{2}{}^{16}O_{7}$ is theoretically 0.01 % of the monoisotopic species. This is measured at a similar level using the Q-MRT instrument and verifies an intra-spectrum dynamic range of at least 10,000 s^{-1}, i.e. four orders, in this case.

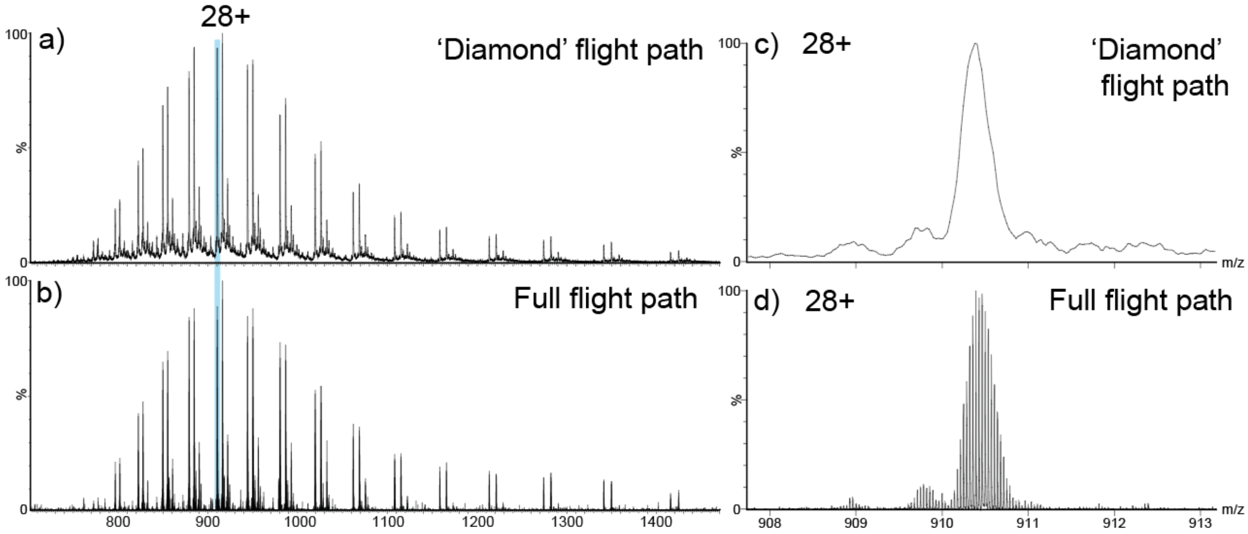

Figure S5. A comparison of the 'diamond' and full flight path modes of operation. a) and b) show the full charge state distributions of the Fc/2 subunit of IdeS-digested NISTmAb in diamond and full flight path modes, respectively. c) and d) show the 28+ charge state of a single isoform (G0F) showing resolving powers of the order 10,000 and 200,000 FWHM, respectively.

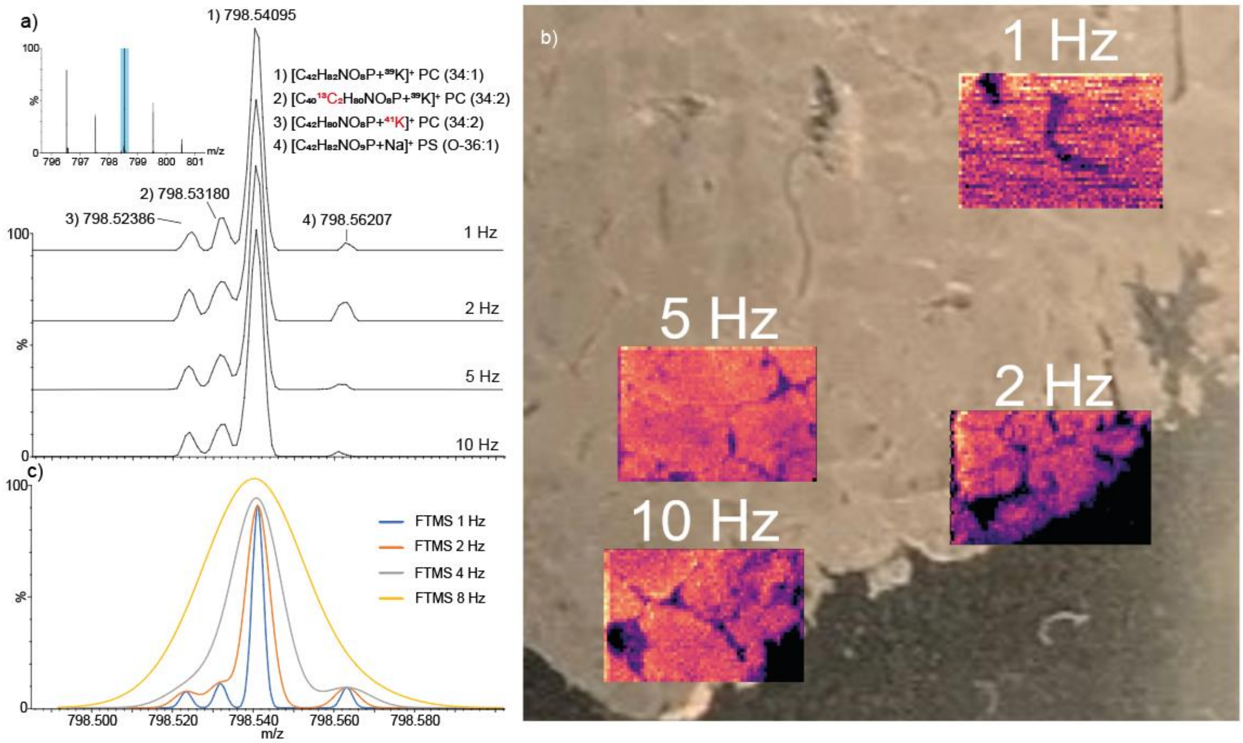

Figure S6. The capabilities of the MRT analyzer for MSI. a) - Zoomed region of the mass spectrum acquired at 1, 2, 5 and 10 Hz by DESI from a section of porcine liver. Four signals are observed some of which correspond to fine isotope structure of nearby lipids. The resolving power does not deteriorate with acquisition speeds. The inset at the top shows a wider, 5 Th, region of the same spectrum. b) The ion images for mass 798.54095 m/z at 1, 2, 5, and 10 Hz. Bottom - a simulated mass spectrum of the same group of signals obtained from a high-field D20 orbitrap mass analyzer at 1, 2, 4, and 8 Hz. The decrease in resolving power as speed is increased is apparent.

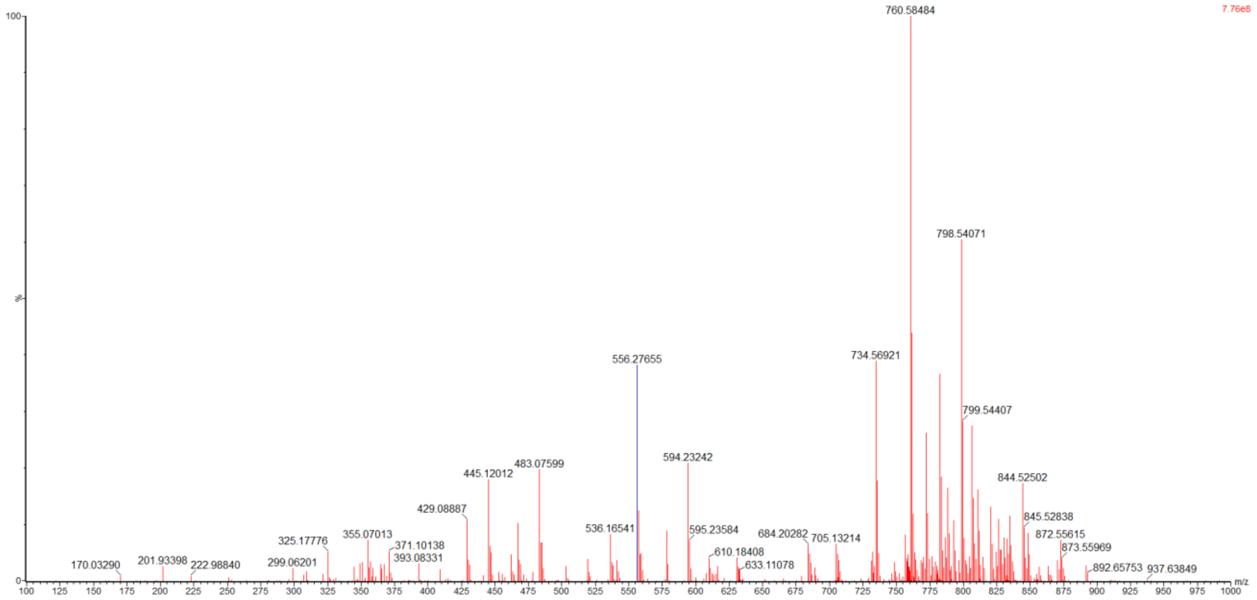

Figure S7. Positive ion DESI mode spectrum from mouse brain tissue

Table S1. Mass accuracy in positive ion DESI mode

Putative ID	Formula	Adduct	Expected mass	Observed mass	mDa error	ppb error
Cholesterol -H2O	$C_{27}H_{44}$	H^+	369.351578	369.35175	0.172	466
LysoPC (16:0)	$C_{24}H_{50}NO_7P$	H^+	496.339766	496.33994	0.174	351
LysoPE (22:6)	$C_{27}H_{44}NO_7P$	H^+	526.292816	526.29291	0.094	179
C18 Sphingomyelin	$C_{41}H_{83}N_2O_6P$	H^+	731.606151	731.60590	-0.251	-343
PC (32:1)	$C_{40}H_{78}NO_8P$	H^+	732.553781	732.55347	-0.311	-425
PC (32:0)	$C_{40}H_{80}NO_8P$	H^+	734.569431	734.56921	-0.221	-302
PC (32:0)	$C_{40}H_{80}NO_8P$	Na^+	756.551376	756.55115	-0.226	-299
PC (34:2)	$C_{42}H_{80}NO_8P$	H^+	758.569431	758.56915	-0.281	-371
PC (34:1)	$C_{42}H_{82}NO_8P$	H^+	760.585082	760.58484	-0.242	-318
PC (32:0)	$C_{40}H_{80}NO_8P$	K^+	772.525313	772.52502	-0.293	-379
PC (34:2)	$C_{42}H_{80}NO_8P$	Na^+	780.551376	780.55176	0.384	492
PC (36:1)	$C_{44}H_{86}NO_8P$	H^+	788.616382	788.61609	-0.292	-370
PC (34:2)	$C_{42}H_{80}NO_8P$	K^+	796.525313	796.52496	-0.353	-443
PC (34:1)	$C_{42}H_{82}NO_8P$	K^+	798.540963	798.54071	-0.253	-317
PC (36:4)	$C_{44}H_{80}NO_8P$	Na^+	804.551376	804.55121	-0.166	-206

PC (38:6)	$C_{46}H_{80}NO_8P$	H^+	806.569431	806.56909	-0.341	-423
PC (36:4)	$C_{44}H_{80}NO_8P$	K^+	820.525313	820.52502	-0.293	-357
PC (36:3)	$C_{44}H_{82}NO_8P$	K^+	822.540963	822.54059	-0.373	-454
PC (36:2)	$C_{44}H_{84}NO_8P$	K^+	824.556613	824.55621	-0.403	-489
PC (36:1)	$C_{44}H_{86}NO_8P$	K^+	826.572263	826.57190	-0.363	-440
PC (38:6)	$C_{46}H_{80}NO_8P$	Na^+	828.551376	828.55103	-0.346	-417
PC (37:6)	$C_{45}H_{78}NO_8P$	K^+	830.509663	830.50940	-0.263	-317
PC (38:5)	$C_{46}H_{82}NO_8P$	Na^+	830.567026	830.56726	0.234	282
PC (40:6)	$C_{48}H_{84}NO_8P$	H^+	834.600732	834.60034	-0.392	-469
PC (38:6)	$C_{46}H_{80}NO_8P$	K^+	844.525313	844.52502	-0.293	-347
PC (38:4)	$C_{46}H_{84}NO_8P$	K^+	848.556613	848.55627	-0.343	-405
PC (40:6)	$C_{48}H_{84}NO_8P$	Na^+	856.582676	856.58240	-0.276	-322
PC (44:12)	$C_{52}H_{80}NO_8P$	H^+	878.569431	878.56909	-0.341	-388
				Mean	-0.209	-244
				SD	0.214	299
				RMS	0.299	**386**

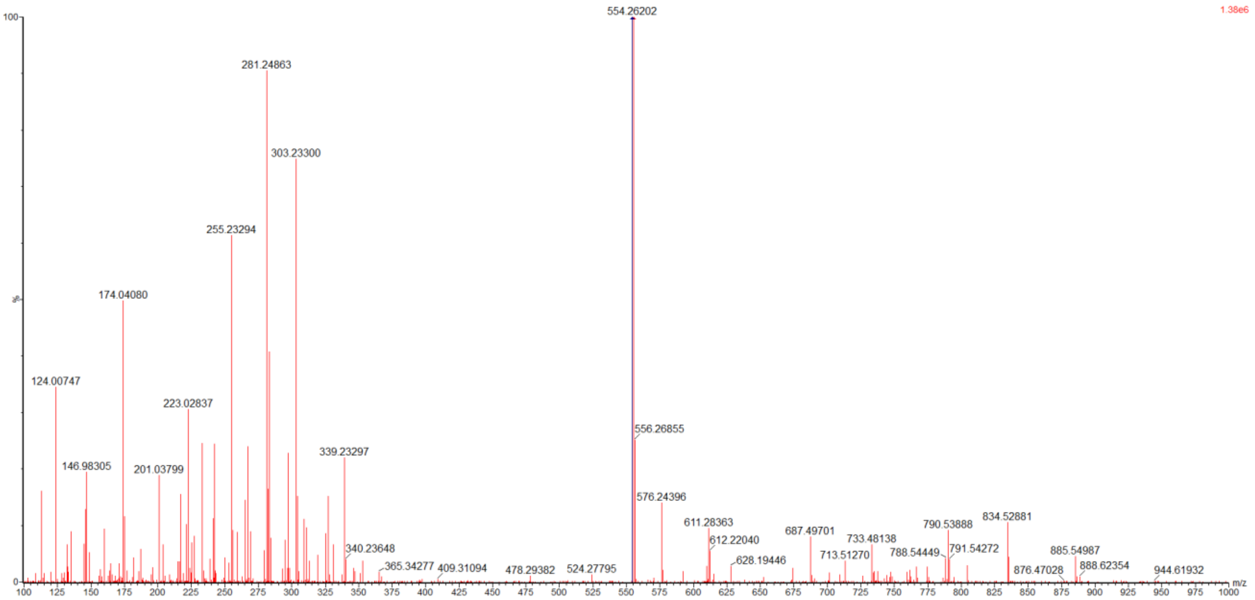

Figure S8. Negative ion DESI mass spectrum from mouse brain tissue

Table S2. Mass accuracy in negative ion DESI mode

Putative ID	Formula	Adduct	Expected mass	Observed mass	mDa error	ppb error
Fumarate	$C_4H_4O_4$	H^-	115.003682	115.00374	0.058	504
Succinate	$C_4H_6O_4$	H^-	117.019332	117.01936	0.028	239
Taurine	$C_2H_7NO_3S$	H^-	124.007387	124.00747	0.083	669
Pyroglutamate	$C_5H_7NO_3$	H^-	128.035317	128.03540	0.083	648
Aspartate	$C_4H_7NO_4$	H^-	132.030231	132.03026	0.029	220
Malate	$C_4H_6O_5$	H^-	133.014247	133.01433	0.083	624
Hypoxanthine	$C_5H_4N_4O$	H^-	135.031234	135.03130	0.066	489
Glutamine	$C_5H_{10}N_2O_3$	H^-	145.061866	145.06187	0.004	28
Glutamate	$C_5H_9NO_4$	H^-	146.045881	146.04590	0.019	130
N-acetyl-Aspartate	$C_6H_9NO_5$	H^-	174.040796	174.04080	0.004	23
Propanetricarboxylic acid	$C_6H_8O_6$	H^-	175.024812	175.02475	-0.062	-354
Palmitoleic acid	$C_{16}H_{30}O_2$	H^-	253.217304	253.21732	0.016	63
Palmitic acid	$C_{16}H_{32}O_2$	H^-	255.232954	255.23294	-0.014	-55
Inosine	$C_{10}H_{12}N_4O_5$	H^-	267.073493	267.07349	-0.003	-11
Linoleic acid	$C_{18}H_{32}O_2$	H^-	279.232954	279.23303	0.076	272
Octadecenoic acid	$C_{18}H_{34}O_2$	H^-	281.248604	281.24863	0.026	92
Octadecanoic acid	$C_{18}H_{36}O_2$	H^-	283.264254	283.26428	0.026	92
Arachidonic acid	$C_{20}H_{32}O_2$	H^-	303.232954	303.23300	0.046	152
Eicosenoic acid	$C_{20}H_{38}O_2$	H^-	309.279904	309.27991	0.006	19
HETE	$C_{20}H_{32}O_3$	H^-	319.227868	319.22794	0.072	226
Adrenic acid	$C_{22}H_{36}O_2$	H^-	331.264254	331.26431	0.056	169
LysoPE (18:1)	$C_{23}H_{46}NO_7P$	H^-	478.293913	478.29382	-0.093	-194
LysoPE (22:6)	$C_{27}H_{44}NO_7P$	H^-	524.278263	524.27795	-0.313	-597
PA (35:1)	$C_{38}H_{73}O_8P$	H^-	687.497029	687.49701	-0.019	-28
PE (P-36:2)	$C_{41}H_{78}NO_7P$	H^-	726.544314	726.54437	0.056	77
PE (P-38:6)	$C_{43}H_{74}NO_7P$	H^-	746.513014	746.51257	-0.444	-595
PC (35:4)	$C_{43}H_{78}NO_8P$	H^-	766.539229	766.53900	-0.229	-299

PE (P40:6)	$C_{45}H_{78}NO_7P$	H^-	774.544314	774.54376	-0.554	-715
PC (37:6)	$C_{45}H_{78}NO_8P$	H^-	790.539229	790.53888	-0.349	-441
PS (37:0)	$C_{43}H_{84}NO_{10}P$	H^-	804.576008	804.57574	-0.268	-333
PS (40:6)	$C_{46}H_{78}NO_{10}P$	H^-	834.529058	834.52881	-0.248	-297
PI (38:4)	$C_{47}H_{83}O_{13}P$	H^-	885.549853	885.54987	0.017	19
				Mean	-0.054	26
				SD	0.168	356
				RMS	0.177	**357**

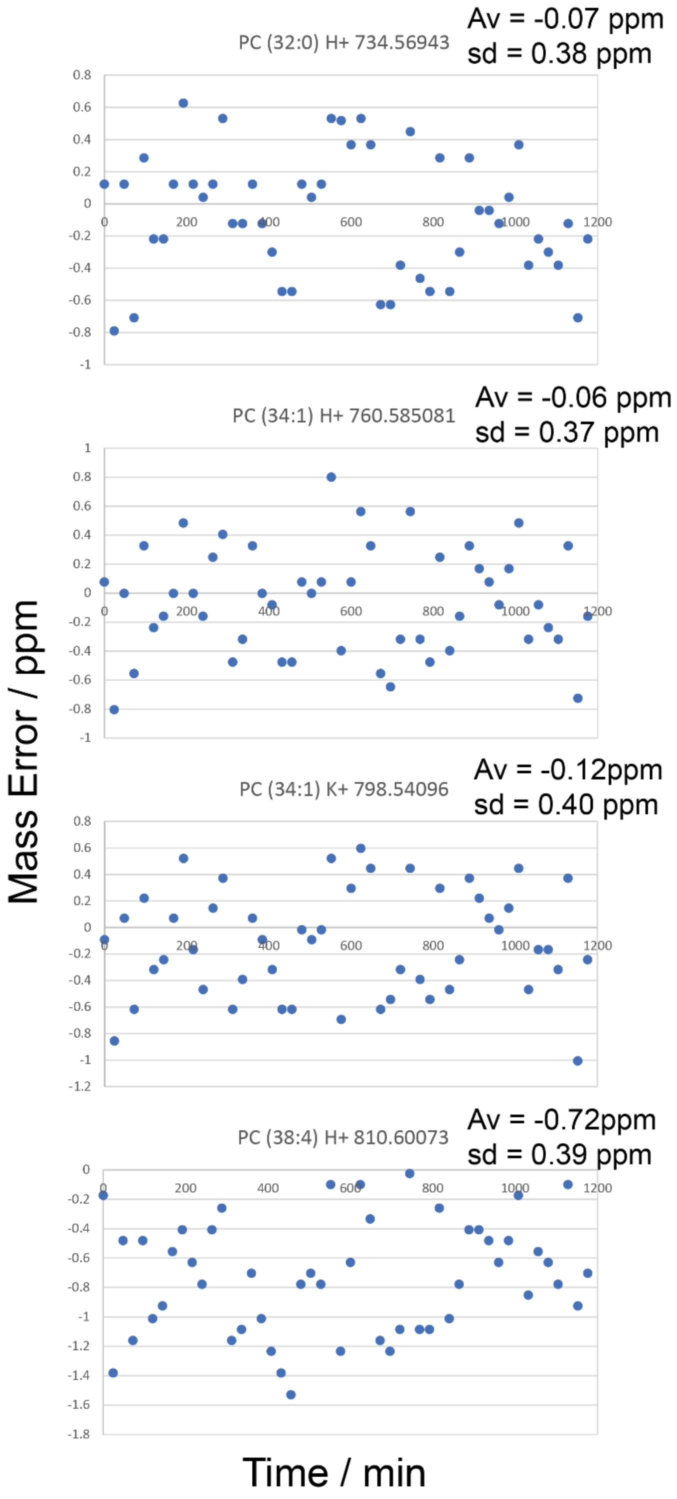

Figure S 9. Mass accuracy for selected evenly-distributed lipids across a DESI-MSI experiment performed on murine brain. Values for PC (32:0) [M+H]+, PC (34:1) [M+H]+, PC (34:1 [M+K]+ and PC (38:4) [M+H]+ are shown for 50 spectra across the 21 hour run after internal lockmass correction (leucine enkephalin). Average and standard deviation values are displayed on each plot. Each lipid has valued scattered around zero except for PC (38:4), suggesting an interference.

Supplementary Methods

Comparisons to Orbitrap instruments

The MRT analyzer is compared in Figure S6 to the D20 high-field compact orbitrap analyzer. At scan rates of approximately 1, 2, 4, and 8 Hz we have assumed transient lengths of 1024, 512, 256, and 128 ms. The spectra were calculated using the enviPat web application[1].

(1) Loos, M.; Gerber, C.; Corona, F.; Hollender, J.; Singer, H. Accelerated Isotope Fine Structure Calculation Using Pruned Transition Trees. *Anal. Chem.* **2015**, *87* (11), 5738–5744. https://doi.org/10.1021/acs.analchem.5b00941.
